# Supplementary material for: Prioritizing public health? Factors affecting the issuance of stay-at-home orders in response to COVID-19 in Africa
Source: PLOS Glob Public Health. 2022 Jan 5;2(1):e0000112. doi: 10.1371/journal.pgph.0000112 (PMC10021525; doi:10.1371/journal.pgph.0000112)
Supplement: Attachment — Submitted filename: PLOSGlobalHealth_R&Rmemo_21-11-06.docx [file pgph.0000112.s001.docx]

PLOS Global Public Health

**Prioritizing Public Health?**

**Factors Affecting the Issuance of Stay-at-Home Orders in Response to COVID-19 in Africa**

Response to Reviewers

Thank you for your interest in this manuscript and the time and effort you invested in your comments to improve it. We share your interest in publishing the best possible research. We appreciate the comments and have made our best effort to address them. Thank you for helping us to significantly improve the paper. Please see next our responses to each comment.

***Actual timeframe covered (paragraph 1 of comments):*** Thank you for noting the discrepancy. Yes, we say six months at places in the manuscript, but, indeed, the time period analyzed is five months. We have revised this in the manuscript (see p. 3). ~Page 13 also states: “June 7 is 30 days after the last African country issued a SAHO during the first wave of the pandemic.”

***Rationale for ending the event history on June 7 (paragraph 1):*** As noted in the manuscript, it is important that the timeframe is theoretically meaningful (Box-Steffensmeier & Jones, 1997). The question regarding the termination of the event history was how much time to include after the last issuance of a stay-at-home order (SAHO). The 41 issuing countries acted somewhat sequentially. The Life Table presented in Appendix A shows that once the first country issued a SAHO, the mean time between the remaining countries to issue a SAHO was 2.7 days with a median of 1 day. The maximum number of days between issuances was 18 days (April 18 to May 6). We chose one month (30 days) with the idea that one month is a discrete and conventional timeframe that would not appear to be selected to bias or p-hack the results by being too short as to miss later issuances but too long as to confound the effects of time. Further, we note that none of the countries that refrained from issuing a SAHO during the event history later issued a SAHO in 2020.

An alternative approach is to terminate the event history after the same number of days that it took for the first issuance of a SAHO after January 31, which is 34 days. That is, the event history would include 34 days before the first issuance and 34 days after the last issuance. This adds an additional four days to the event history. Thirty-four days after May 8 is June 11. Re-estimating the main model, Model 1, with this timeframe generates very similar results.

```
Probit regression                               Number of obs     =      4,190
                                                Wald chi2(13)     =     118.92
                                                Prob > chi2       =     0.0000
Log pseudolikelihood = -176.06838               Pseudo R2         =     0.2361

-------------------------------------------------------------------------------
              |               Robust
sahoStatusEHA |      Coef.   Std. Err.      z    P>|z|     [95% Conf. Interval]
--------------+----------------------------------------------------------------
  regTypeFH_n |
  1/partFree  |     -0.617      0.202    -3.06   0.002       -1.013      -0.222
      2/free  |     -0.578      0.313    -1.85   0.064       -1.191       0.034
              |
  fragility_c |     -0.036      0.029    -1.21   0.226       -0.093       0.022
              |
    colLeg_n2 |
    2/France  |      0.064      0.187     0.34   0.733       -0.303       0.431
   3/neither  |     -0.128      0.185    -0.69   0.490       -0.490       0.234
              |
 govHlthSpd_c |      0.007      0.007     0.88   0.380       -0.008       0.021
   urbanPop_c |     -0.006      0.005    -1.09   0.278       -0.016       0.004
gdpPerCapLn_c |      0.104      0.111     0.94   0.347       -0.113       0.321
      casesMD |      0.248      0.044     5.65   0.000        0.162       0.334
ebolaCasesDum |      0.609      0.226     2.70   0.007        0.167       1.051
 prepStatus_c |     -0.046      0.140    -0.33   0.743       -0.319       0.228
      geoDiff |     -0.003      0.003    -0.89   0.376       -0.010       0.004
   tempDiff66 |      0.001      0.000     7.01   0.000        0.000       0.001
        _cons |     -3.253      0.212   -15.35   0.000       -3.668      -2.838
-------------------------------------------------------------------------------
```

We believe the original timeframe is most defensible because it is not ad hoc, and it yields very similar estimates. Further, we provided additional justification on p. 13:

*The decision to terminate the event history 30 days after the last issuance is based on a number of considerations. None of the countries that refrained from issuing a SAHO to that point later issued a SAHO in 2020. One month is a discrete and conventional timeframe. It balances the need to give reasonable time to capture additional issuances (the mean [median] time between issuances was 2.7 [1] days and the maximum was 18 days) with the need to avoid confounding the effects of time by selecting an overextended timeframe.*

***Insufficient theoretical frame/more careful discussion of hypotheses (paragraph 2 and 3):*** We have extensively revised the paper with additional theory. Specific to the Africa Policy Context section, we added a full paragraph regarding Ebola and the response to it (see ~pp. 6-7). Overall, though, we added more theory, in particular in the Literature and Hypotheses section, to support the hypotheses.

The additional literature includes: Akanle (2013), Alesina et al. (2003), Cohen (1970), Gasiortowski (2016), Hinnebusch & Ehteshami (2002), Kieh (2013), King (2009), Lagi et al. (2011), Maher & Sekajugo (2011), Maseland (2018), Maxmen (2020), Meltsner (1972), Oliver (2006), Stapleton (2019), Theme-Filha (2005), and Zoubir (2015).

***One-size-fits-all statements (paragraph 2):*** In general, we have extensively revised the Africa Policy Context section, in particular attempting to emphasize the heterogeneity of the continent. The second and third paragraphs discuss the differing regions (see ~pp.4-5 starting “However, not all Africa confronts...” and “The rest of the continent...”). Further, to address comments regarding regions, we added a regional variable to the multivariate models (e.g., see p. 17). The regions are specified by UN Stats and include Northern (comparison group), Eastern, Middle, Southern, and Western Africa. We also report the results for when the regional variable is replaced with an indicator variable for Sub-Saharan Africa (1) or not (0).

In regard to public health specifically, we have also added a comment and figure (Figure 1) indicating the variation in Africa regarding pandemic preparedness.

***Hypothesis 3b and socio-demographics (paragraph 4):*** To clarify the idea of risk, we add on p. 11:

*Vulnerability to disease varies, for instance, by age, sex, ethnicity, education, income, and marital status (e.g., Theme-Filha et al., 2005). As such, government policies vary to account for variations in the populations they serve.......It is likely, then, that the populations of some countries are more vulnerable than the populations of other countries."*

Further, population density seems to be particularly relevant to research regarding infectious disease, but it also makes sense to capture other socio-demographic effects as suggested in the comments. As such, we also tested effects related to ethnic fractionalization (Alesina et al., 2003), religious fractionalization (Alesina et al., 2003), net migration, years of compulsory education, and percent population 65 years old and older. As noted on p. 21, none of these other measures are associated with statistically significant effects.

***Temporal policy diffusion (paragraph 5):*** We added substantial new information regarding this issue. We added references to related policy literature in the Literature and Hypotheses section (see about p. 13). We also added supporting information in the results, particularly a figure (Figure 2), that presents the actual shape of the data (see ~pp. 21-22), which supports the S-shaped expectation.

***Health policy transition in Africa (paragraph 5):*** This issue makes sense, and we briefly address it now on p. 13. That said, we believe longer-term forces such as this and some of the socio-demographic affects are overwhelmed by the urgent nature of the pandemic. We support this argument by noting in the Conclusion that all the SAHO issuances happened within a 64-day period (see p. 25).

***Results section and deeper theory (paragraph 6):*** As noted above, we added more theory, in particular in the Literature and Hypotheses section, to support each hypothesis. We made a special effort in the Conclusion to add greater theoretical context to each of the findings and overall including expanded discussion of the results. We also offered a more thorough grand summary of what we believe happened (see p. 25):

*Overall, the results, particularly from Table 1 regarding relative effects, suggest that medical considerations were a priority for decision makers, but that temporal diffusion, an external factor, played the most significant role. On the other hand, the evidence is mixed at best regarding the effect of political considerations and clear that social and economic factors played little to no role. The lack of effects for some factors, particularly the social and economic factors, may be related to the speed with which the SAHOs were issued. The 41 countries that locked down issued their SAHOs within a 64-day period. The quickly mounting worldwide cases and deaths drew significant public attention, which may have pushed the pandemic into the realm of Christensen et al.’s (2016) “wicked” crises composed of transboundary, unique, and uncertain threat, which made an accelerated response rational (Nicholson-Crotty, 2009). Over such an abbreviated and anxiety provoking period of time, it is likely that longer-term factors, such as social and economic issues, would be uncertain and incompletely understood relative to the medical considerations and, therefore, devalued relative to the medical considerations.*

***Miscellaneous by authors***

We noted that casesMD is highly positively skewed (see the table and figure below), and no typical transformation sufficiently normalized the skew. To meet the normality assumption of linear regression, we transformed the variable into a series of four indicator variables (see pp. 19-20). Variable 1 is coded 1 when there are 0 cases per MD and 0 otherwise (n=2542). The remaining three variables are based on an approximately equal distribution of the remaining observations (532, 533, and 531, respectively), which occurred when there were detected COVID cases in a country. Variable 2 is coded 1 when there are .0000134 to .0068234 cases per MD and 0 otherwise (n=532). Variable 3 is coded 1 when there are .0068742 to .1079812 cases per MD and 0 otherwise (n=533). Variable 4 is coded 1 when there are .1088889 to 15.81818 cases per MD and 0 otherwise (n=531). It should be noted that the results regarding this measure and overall model are quite similar regardless of which form of this variable is used.

```
. sktest casesMD if e(sample)

                    Skewness/Kurtosis tests for Normality
                                                          ------ joint ------
    Variable |        Obs  Pr(Skewness)  Pr(Kurtosis) adj chi2(2)   Prob>chi2
-------------+---------------------------------------------------------------
     casesMD |      4,138     0.0000        0.0000           .              .
```

For completeness, we added bivariate analyses to identify the zero-order correlations of the independent variables with the dependent variable. See the first paragraph of the Results section (p. 18) and Appendix D.

Thank you.
